# Pediatric Voluntary Habitual Hip Dislocation: Clinical Characteristics, Family Dynamics, and Long-Term Outcomes—A Retrospective Study

**DOI:** 10.3390/jcm14031022

**Published:** 2025-02-06

**Authors:** Mehmet Yılmaz, İbrahim Ulusoy, Mehmet Fırat Tantekin, İsmail Güzel, Aybars Kıvrak

**Affiliations:** 1 Department of Orthopedics and Traumatology, Gaziantep City Hospital, 27470 Gaziantep, Türkiye; 2 Department of Orthopedic Surgery, Selahaddin Eyyubi State Hospital, 21100 Diyarbakır, Türkiye; dr.ibrahimulusoy@gmail.com (İ.U.); firatantekin@hotmail.com (M.F.T.); 3 Department of Orthopedics and Traumatology, Turgut Özal University, 44210 Malatya, Türkiye; dr.ismailguzel@gmail.com; 4 Department of Orthopedic Surgery, Adana Avrupa Hospital, 01170 Adana, Türkiye; aybarskivrak@gmail.com

**Keywords:** voluntary habitual dislocation, pediatric hip dislocation, family dynamics, conservative management, posterior hip dislocation, treatment outcomes, psychosocial factors

## Abstract

**Background/Objectives:** Recurrent hip dislocations are a rare occurrence in pediatric patients. As there are few cases of voluntary habitual dislocation documented in the literature, there is a paucity of information available regarding its pathogenesis, risk factors, and classification. The prognosis for these patients is generally good. A long-term follow-up duration of two years was conducted to evaluate outcomes. Statistical analysis was performed to assess the impact of family structure and treatment approaches on outcomes. **Methods:** From January 2010 to December 2022, patients with voluntary habitual hip dislocation were retrospectively identified through the hospital information system. Data regarding demographic characteristics, clinical findings, and treatment outcomes were analyzed. A total of 13 patients (14 hips) met the inclusion criteria. Conservative treatment methods, including orthosis and family therapy, were applied. Statistical analysis was performed to assess the impact of family structure and treatment approaches on outcomes. **Results:** The mean age at diagnosis was 48.7 months, with 77% of cases being female. Posterior dislocation was observed in all cases, and no underlying bone pathology was detected on imaging. The prognosis for these patients is generally good. At the one-year follow-up, 85% of patients achieved a complete resolution of dislocations, increasing to 100% by the two-year follow-up. Patients from larger families demonstrated significantly slower recovery rates at the first- and sixth-month evaluations (*p* = 0.033 and *p* = 0.048, respectively), but outcomes were comparable by one year. A unique aspect of this study is the emphasis on family dynamics, which significantly influenced treatment adherence and recovery. Statistical analysis was performed to assess the impact of family structure and treatment approaches on outcomes. **Conclusions:** Voluntary habitual hip dislocation is a rare condition with good long-term outcomes under conservative management. This study highlights the importance of addressing family dynamics in the treatment plan, especially in larger families, where attention and psychological factors may play a significant role in delayed recovery.

## 1. Introduction

Recurrent hip dislocations are a rare phenomenon in pediatric patients, often associated with underlying pathological conditions such as trauma sequelae, connective tissue disorders (e.g., Ehlers–Danlos syndrome), bone dysplasias, or genetic conditions like Down syndrome [1,2]. However, in rare instances, hip dislocations occur voluntarily and habitually, without any identifiable underlying pathology. This condition is referred to as voluntary habitual dislocation and is poorly understood due to its infrequent reporting in the literature [3,4].

The pathogenesis of voluntary habitual dislocation remains unclear. It is hypothesized that psychological or behavioral factors may play a role, as the dislocation is often performed intentionally by the child, typically in positions involving hip flexion, adduction, and internal rotation [5]. This theory is supported by Faienza et al. who suggested that voluntary dislocations may be a coping mechanism for children facing psychosocial stressors, as the act of dislocating may provide a sense of control or attention [6]. Furthermore, Ricciuti et al. highlighted that the absence of pain in most cases, as observed in our study, reinforces the behavioral nature of the dislocation, distinguishing it from traumatic or pathological dislocations [7]. Despite the lack of structural abnormalities, the dislocation is usually painless, and patients maintain a normal gait, with a negative Trendelenburg test [8].

The nomenclature of this condition is inconsistent in the literature, with terms such as “voluntary recurrent”, “habitual voluntary”, and “recurrent habitual” being used interchangeably. This variability reflects the limited understanding of its etiology and classification [9]. Ambrus et al. emphasized that standardizing terminology is critical for improving diagnostic consistency and facilitating comparative studies across populations [10]. Treatment approaches vary widely, ranging from conservative management with orthosis and psychiatric therapy to more invasive interventions, though surgery is rarely indicated [10,11]. A systematic review by Caliesch et al. demonstrated that conservative treatments are generally effective, particularly when behavioral therapies are integrated, further supporting their role as first-line management for voluntary habitual dislocations [12].

Despite the conservative management approaches commonly utilized for voluntary habitual hip dislocation, there is a lack of standardized diagnostic and treatment protocols. The inconsistent nomenclature and limited understanding of the pathogenesis further complicate diagnosis and management. Existing studies primarily focus on case reports or small cohorts, which do not adequately address the role of family dynamics or behavioral interventions. Additionally, there are limited data on the long-term efficacy of conservative treatments, particularly in socio-culturally diverse populations. Our study aims to fill these gaps by providing a comprehensive analysis of the clinical characteristics, family dynamics, and treatment outcomes in a larger cohort with long-term follow-up.

In this study, we aimed to analyze 13 new cases of voluntary habitual dislocation to provide clinical insights into this rare condition and to identify factors affecting treatment outcomes. Furthermore, we reviewed the existing literature to contextualize our findings and to contribute to the understanding of this unique pathology.

## 2. Materials and Methods

### 2.1. Study Design and Study Population

This retrospective observational study was conducted to evaluate pediatric patients diagnosed with voluntary habitual hip dislocation between January 2010 and December 2022. Ethical approval was obtained from the Clinical Research Ethics Committee of Dicle University (approval number: 212-316), and all procedures were performed in accordance with the Declaration of Helsinki.

To minimize recall bias inherent to the retrospective design, all data were extracted from a centralized hospital information management system, which includes detailed and standardized medical records. These records documented patient demographics, clinical findings, imaging results, and follow-up evaluations using consistent templates across all included cases. Additionally, treatment protocols for voluntary habitual hip dislocation, including orthosis and family therapy, were guided by institutional guidelines, ensuring uniformity in patient management. The availability of detailed and structured records reduced variability and enhanced the reliability of the extracted data.

Inclusion criteria were designed to ensure a homogenous sample by selecting pediatric patients under 18 years of age with a diagnosis of voluntary habitual hip dislocation confirmed through clinical evaluations and imaging. Patients were required to have a minimum follow-up period of two years to allow for a comprehensive assessment of treatment outcomes. Patients with connective tissue disorders, such as Ehlers–Danlos syndrome or Marfan syndrome, were excluded based on clinical evaluations and available medical history. Diagnostic criteria for these conditions, including joint hypermobility, skin elasticity, and other systemic manifestations, were assessed during initial evaluations. Genetic testing or advanced diagnostic evaluations, such as echocardiography or molecular analyses, were not routinely performed in this study due to the retrospective design and the absence of clinical indications in the included cohort. Patients with any prior clinical diagnosis of connective tissue disorders or significant phenotypic findings suggestive of such conditions were excluded to ensure the homogeneity of the study population.

Clinical evaluations included the documentation of age, gender, family structure (e.g., the number of siblings), the type of dislocation (unilateral or bilateral), provocative positions, and symptom severity (painful or painless). Imaging studies primarily consisted of plain radiographs to assess hip joint positioning. Computed tomography (CT) scans were performed to exclude underlying bone pathologies, though magnetic resonance imaging (MRI) during dislocation events was not feasible due to the voluntary and transient nature of the condition. Despite this limitation, standard imaging in the neutral position revealed no structural abnormalities.

Families were grouped based on the number of children (<3 children vs. ≥3 children) to investigate the potential impact of family size on treatment adherence and recovery outcomes. This classification was guided by prior research suggesting that larger family sizes may influence parental attention, caregiving resources, and overall psychosocial dynamics, which are known to affect pediatric health outcomes [13,14]. The threshold of three children was selected as it aligns with sociocultural norms in the study region and represents a meaningful division in terms of resource distribution and family dynamics.

Conservative treatment methods were employed for all patients, including the use of an abduction orthosis and therapy sessions with a child psychiatrist, often accompanied by family therapy. The effectiveness of these interventions was evaluated at regular follow-up intervals of one, six, and twelve months, as well as at two years. Clinical outcomes were assessed based on the frequency of dislocation, patient-reported symptoms such as pain and functional mobility, and radiographic findings. To assess generalized joint laxity, the Beighton hypermobility score was utilized in all patients.

Dynamic imaging, such as MRI or fluoroscopy during dislocation events, was not feasible in this study due to the voluntary and transient nature of the dislocations. These dislocations were self-induced and typically resolved immediately after the provoking position was released, making it challenging to capture the exact moment of dislocation. Additionally, ethical considerations regarding radiation exposure in pediatric patients further limited the use of fluoroscopy.

The Beighton hypermobility score was administered by a trained study nurse under the supervision of a senior orthopedic specialist. The scoring involved evaluating joint flexibility at nine anatomical sites, including bilateral knees, elbows, and fingers, as well as the spine. Each site was assessed based on the standard criteria, with the total score ranging from 0 to 9. A score of 4 or higher typically indicates hypermobility. The assessments were conducted in a controlled clinical setting to ensure consistency and reliability.

### 2.2. Statistical Analysis

Statistical analyses were conducted using SPSS Statistics version 25.0 (IBM Corp., Armonk, NY, USA). Descriptive statistics were used to summarize demographic data and treatment outcomes, with continuous variables presented as mean ± standard deviation or median with interquartile ranges, depending on data normality (assessed by Kolmogorov–Smirnov and Shapiro–Wilk tests). To evaluate the association between family size and treatment outcomes, univariate analyses (Chi-square test for categorical variables and independent t-tests or Mann–Whitney U tests for continuous variables) were performed. Additionally, multivariate logistic regression analysis was conducted to adjust for potential confounding variables, such as age, gender, and initial clinical presentation, in assessing the impact of family dynamics on treatment outcomes. A *p*-value of less than 0.05 was considered statistically significant.

## 3. Results

Patient demographics, treatment methods, and follow-up outcomes are shown in Table 1. Following a comprehensive retrospective evaluation, the medical records of 39 patients diagnosed with hip dislocations were reviewed. Under the inclusion and exclusion criteria, 13 patients (14 hips) were included in the study cohort. The mean age of the patients at the time of diagnosis was 48.7 ± 6.3 months, with a range of 39 to 63 months. The majority of patients were female (77%, *n* = 10), while three patients (23%) were male. Among the affected hips, 10 were on the right side (71.4%) and 4 were on the left (28.6%), and one patient exhibited bilateral hip involvement. All dislocations observed were posterior, with no cases of anterior dislocation identified. Clinically, 12 patients experienced painless dislocations, while 1 patient reported occasional pain during dislocation episodes. The provocative positions for dislocation included hip flexion, adduction, and internal rotation in all cases. None of the patients displayed signs of gait abnormalities, and the Trendelenburg test results were negative in all cases. Additionally, the Beighton hypermobility score was zero for all patients, indicating an absence of generalized joint laxity (Table 1).

Table 1 also summarized the treatment outcomes across all follow-up intervals. Overall, 11 of the 13 patients achieved a complete resolution of dislocations by the one-year follow-up, while the remaining 2 patients continued to experience occasional dislocations at the one-year mark but showed improvement by the two-year follow-up. The subgroup analysis suggested that the slower response to treatment in families with more children might be attributed to reduced parental attention and increased psychological stress in these family settings (Table 1).

**Figure 1:**
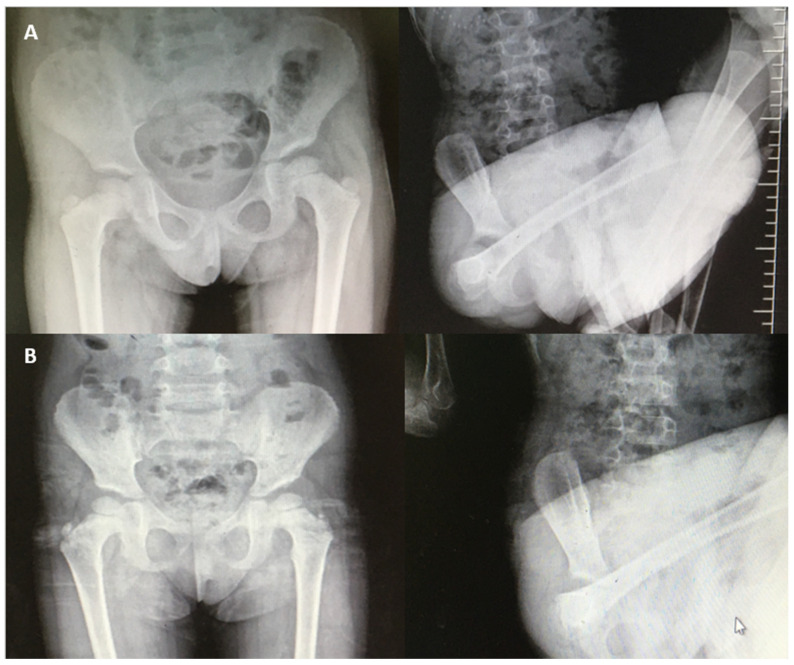
(**A**) Radiographs showing normal and dislocated hip positions in a 46-month-old female patient. (**B**) Radiographs showing normal and dislocated hip positions in a 54-month-old female patient.

The imaging findings were consistent across the cohort. While computed tomography (CT) scans of the hips in the neutral position showed no structural abnormalities, imaging during dislocation events was not feasible. However, radiographic evaluations confirmed that all dislocations were purely positional, without associated osseous deformities or pathologies. No adverse events or complications were reported during the follow-up period. The conservative management approach demonstrated high efficacy in this cohort, with a favorable long-term prognosis. Figure 1 shows representative radiographs of a patient at the time of dislocation and subsequent reduction in the hip. These findings highlight the importance of considering family dynamics in the treatment plan for voluntary habitual hip dislocations and emphasize the role of multidisciplinary management, including family therapy, in achieving optimal outcomes.

Conservative management was the primary treatment modality for all patients. This included the use of an abduction orthosis combined with child and family therapy sessions provided by a child psychiatrist. The response to treatment was assessed at one, six, and twelve months, as well as at two years post treatment. Families were stratified into two groups based on the number of children: fewer than three children (Group 1) and three or more children (Group 2). The response to treatment was significantly lower in families with three or more children at the first- and sixth-month evaluations, with *p*-values of 0.033 and 0.048, respectively. However, no significant differences were observed at the one-year and two-year follow-ups (*p* > 0.05) (Table 2).

Multivariate logistic regression analysis confirmed that family size (≥three children) was independently associated with slower treatment response at the first- and sixth-month follow-ups (adjusted odds ratio: 3.5; 95% confidence interval: 1.2–10.1; *p* = 0.033). However, no significant association was observed at the one-year and two-year follow-ups (*p* > 0.05). These results underscore the role of family dynamics in early recovery phases.

## 4. Discussion

Voluntary habitual hip dislocation is a rare condition, particularly in pediatric populations, and remains poorly understood due to its infrequent occurrence and the lack of large-scale studies. Our study provides valuable insights into this unique pathology, presenting clinical characteristics, treatment outcomes, and the potential influence of family dynamics on recovery. In this discussion, we compare our findings with the existing literature and explore their implications for clinical practice.

Surgical intervention for voluntary habitual hip dislocation is typically reserved for cases that are refractory to conservative management or associated with structural abnormalities, as noted in the literature. For instance, Zielinski et al. reported that surgical treatment, including soft tissue reconstruction or osteotomy, might be considered in rare cases where dislocations are accompanied by persistent pain or functional impairment despite behavioral therapies [15]. However, the patient population in our study exhibited no structural abnormalities on imaging and responded favorably to conservative treatments, including orthoses and family therapy. This distinction highlights the importance of patient selection in determining the optimal management strategy. Our findings emphasize that, in the absence of underlying anatomical defects, conservative approaches should remain the first-line treatment. Further studies comparing outcomes between surgical and non-surgical cohorts could provide a more comprehensive framework for treatment decision making.

Posterior dislocation was observed in all cases in our study, a finding consistent with prior studies on voluntary habitual hip dislocations. This emphasis is critical as the posterior type appears to be the predominant mechanism in this condition, likely due to the biomechanics of hip flexion, adduction, and internal rotation, which are common provocative positions. Unlike anterior dislocations, posterior dislocations typically occur without structural abnormalities and are often painless, as observed in our cohort. Moon et al. also reported that posterior dislocations dominate in voluntary cases, suggesting a primarily positional etiology rather than one related to anatomical defects [16]. Jacobsen et al. highlighted that voluntary dislocations typically occur in positions of hip flexion, adduction, and internal rotation, as observed in our cohort [17]. These findings suggest that the dislocation mechanism is primarily positional and not associated with intrinsic structural abnormalities. Supporting this, Zielinski et al. conducted a systematic review and noted that the absence of structural abnormalities in imaging is a consistent feature of voluntary habitual hip dislocations, further reinforcing the positional nature of the pathology [15]. Additionally, Brown et al. emphasized that these cases often demonstrate normal hip joint anatomy on radiographs and CT scans, indicating that the dislocations are driven by patient behavior or habitual positioning rather than anatomical defects [18]. This body of evidence collectively underscores that voluntary habitual hip dislocations represent a distinct clinical entity, where the pathophysiology is largely mechanical and behavioral rather than structural.

One of the unique aspects of our study is the investigation of family dynamics and its impact on treatment outcomes. We found that patients from families with three or more children exhibited slower treatment responses during the first and sixth months. Heath et al. emphasized the role of psychosocial factors, including parental attention and family stress, in pediatric musculoskeletal conditions [13]. Kelada et al. demonstrated that reduced parental involvement and increased sibling competition for attention in larger families negatively impact adherence to treatment regimens and overall recovery in children with chronic conditions [14]. Furthermore, Vinall et al. reported that children from larger households are more likely to experience psychological distress, which may manifest as behavioral challenges or delayed responses to therapy in musculoskeletal conditions [19]. In larger families, limited individual attention may contribute to psychological stress in children, potentially delaying treatment responses. This underscores the importance of integrating family therapy into the management plan for such patients, as addressing psychosocial factors can significantly enhance compliance and improve treatment outcomes.

The conservative management approach employed in our study demonstrated high efficacy, with 85% of patients achieving the complete resolution of dislocations by the one-year follow-up. Rakotonandrianina et al. reviewed conservative treatments for voluntary hip dislocations and reported favorable outcomes with the use of orthosis and behavioral therapy [20]. Keating et al. highlighted that these conservative interventions, particularly those incorporating family therapy and orthosis, are effective in addressing voluntary habitual dislocations, with long-term success rates exceeding 80% [21]. Notably, none of our patients required surgical intervention, which further supports the effectiveness of non-invasive strategies in managing this condition. The avoidance of surgical risks and the achievement of successful outcomes reinforce the role of conservative management as a first-line treatment for voluntary habitual hip dislocations.

Although our study focuses on conservative management, the absence of a direct comparison with surgical options represents a significant limitation. Surgical interventions are rarely indicated for voluntary habitual hip dislocation, given its behavioral and positional etiology; however, they may still be considered in severe or refractory cases. Studies such as Keating et al. have highlighted favorable outcomes with conservative approaches, but data comparing these with surgical interventions remain scarce [21]. Including a surgical cohort in future research could provide a more comprehensive understanding of the relative effectiveness of these treatment modalities and inform clinical decision making.

Although our findings are promising, there are some discrepancies when comparing the response rates in larger families to other studies. Knafl et al. did not find significant differences in treatment outcomes based on family size [22]. Sborov et al. suggested that while family dynamics play a role in treatment adherence, the impact of family size may be mitigated by other factors, such as socioeconomic status and parental involvement [23]. In contrast, Pourbordbari et al. observed that larger family size was associated with delayed recovery in pediatric patients with chronic musculoskeletal conditions, citing reduced individual attention and increased caregiver burden as contributing factors [24]. These discrepancies may be attributed to differences in study design, cultural contexts, and the inclusion criteria for voluntary dislocations. For instance, our study focused on a population with unique sociocultural dynamics, including extended family systems and varying parental roles, which might amplify the effects of family size on treatment outcomes. Such findings underscore the need for multicenter studies that account for cultural and socioeconomic variability to validate these observations and refine treatment strategies for voluntary habitual hip dislocations.

Sociocultural factors likely play a significant role in influencing family dynamics and treatment adherence, as suggested by the slower recovery observed in larger families. In our study cohort, extended family systems, common in the study region, may have provided additional caregiving support but also introduced challenges related to shared responsibilities and competing priorities. These dynamics can impact the consistency of treatment adherence, particularly in families with more children. Similar findings were noted in studies by Heath et al. and Vinall et al., which highlighted the role of socioeconomic and cultural factors in shaping parental involvement and pediatric health outcomes [13,19]. Future studies should explore the impact of specific sociocultural variables, such as education level, economic status, and family structure, to better understand their influence on treatment outcomes. Incorporating validated tools to measure these variables could provide deeper insights into their role in the management of voluntary habitual hip dislocation.

Another notable finding was the absence of generalized joint laxity in our cohort, as indicated by negative Beighton scores. Gensemer et al. reported that joint hypermobility was a contributing factor in some cases of habitual dislocation, particularly in patients with underlying connective tissue disorders such as Ehlers–Danlos syndrome [25]. Our findings that voluntary habitual hip dislocation is unrelated to systemic joint laxity contrast with prior studies, such as the work by Rejeb et al., which identified joint laxity as a common factor in recurrent dislocations, particularly among younger patients [26]. These discrepancies may be attributed to differences in study populations and evaluation methods. For instance, Rejeb et al. primarily focused on athletes and individuals with high levels of physical activity, a population inherently predisposed to joint hypermobility due to repetitive stress on the joints. In contrast, our study included a more general pediatric population with no history of high-impact activities, which may explain the absence of generalized joint laxity in our cohort. Additionally, Rejeb et al. utilized a more extensive set of diagnostic tools, including advanced imaging and biomechanical assessments, to evaluate joint characteristics, whereas our study relied on clinical assessments such as the Beighton hypermobility score. This difference in evaluation methods could also account for the lack of subclinical findings in our population. These methodological distinctions underscore the importance of standardizing evaluation protocols across studies to facilitate more accurate comparisons. Future research incorporating both advanced diagnostic techniques and a broader patient demographic could help reconcile these differences and further delineate the unique characteristics of voluntary habitual hip dislocation.

The decision to group families by the number of children was informed by evidence from previous studies, which have shown that larger family sizes can negatively impact treatment adherence and recovery in pediatric populations. Heath et al. reported that families with more children often face challenges related to reduced individual attention and increased caregiver burden [13]. This stratification allowed us to explore the role of family dynamics, particularly in resource-limited settings, in influencing treatment outcomes. Our findings of slower recovery in larger families highlight the importance of targeted family therapy and support in managing voluntary habitual hip dislocation.

While our study stratified families into two groups (fewer than three children vs. three or more children), a direct comparison of single-child families with larger families was not explicitly performed. Such an analysis may be feasible in future studies with a larger sample size, as the current cohort included only one single-child family, limiting statistical power for this subgroup.

Previous studies have also highlighted the influence of family dynamics on treatment adherence and recovery. For example, Heath et al. reported that reduced parental attention and increased stress in larger families negatively impact treatment outcomes in pediatric musculoskeletal conditions [13]. Vinall et al. emphasized that psychological distress in children from larger families may delay recovery, potentially due to competition for parental attention [19]. Our findings align with these observations, suggesting that family size plays a critical role in treatment response, particularly in the early phases of recovery.

The slower recovery observed in patients from larger families may be attributed to several factors. First, limited parental attention in larger families may reduce adherence to treatment protocols, such as the consistent use of orthosis and attendance at therapy sessions. Second, psychological stress due to sibling competition for attention may contribute to delayed behavioral and emotional adjustments necessary for recovery. Lastly, socioeconomic challenges, which are often more pronounced in larger families, may exacerbate these issues by limiting access to resources or increasing caregiver burden.

Our findings suggest that voluntary habitual hip dislocation is a distinct clinical entity unrelated to systemic joint laxity, as all patients in our cohort demonstrated negative Beighton scores. This observation contrasts with other forms of habitual dislocations that are often linked to generalized joint hypermobility, such as those seen in connective tissue disorders like Ehlers–Danlos syndrome [25]. The absence of systemic joint laxity in our cohort may point to a primarily behavioral or positional etiology rather than a structural predisposition. However, it is worth noting that previous studies have suggested potential subclinical connective tissue alterations in some patients without overt hypermobility [26]. Future studies employing advanced imaging techniques or molecular analyses could explore whether subtle biomechanical or histological abnormalities contribute to this unique condition. Additionally, the exclusion of patients with known connective tissue disorders in our study underscores the need for a broader investigation to confirm the distinctiveness of voluntary habitual hip dislocation as a clinical entity.

Dynamic studies, such as fluoroscopy or MRI, could significantly enhance our understanding of the biomechanics underlying voluntary habitual hip dislocation. The transient and voluntary nature of these dislocations poses a challenge for capturing the exact moment of dislocation using standard imaging techniques. However, advanced imaging methods, like those described in the recent literature have demonstrated potential in providing real-time insights into joint mechanics and movement patterns during dislocation events [27]. Incorporating these diagnostic tools into future research could allow for a more detailed exploration of the pathophysiology of the condition, particularly in identifying subtle biomechanical or positional abnormalities that may not be evident on static imaging. Moreover, dynamic imaging may also aid in tailoring individualized therapeutic interventions by pinpointing specific mechanical triggers for dislocation.

### Limitations of the Study

This study has several limitations that must be acknowledged. As a retrospective analysis, it is subject to inherent biases, including recall bias from reliance on medical records and the absence of real-time data collection. The lack of dynamic imaging during dislocation events, such as fluoroscopy or MRI, limited the ability to investigate the precise biomechanics of voluntary habitual hip dislocations. Additionally, the small sample size, reflective of the rarity of the condition, and the single-center design restrict the generalizability of the findings. Sociocultural factors unique to the study region may have influenced family dynamics and treatment adherence, potentially affecting the results. Furthermore, the exclusive focus on conservative treatment without a comparison to surgical interventions limits the scope of management strategies evaluated. Addressing these limitations in future prospective, multicenter studies with larger cohorts and advanced imaging techniques will be critical to enhancing the understanding and management of this rare condition.

The strength of our study lies in its rigorous methodological approach, including the clear definition of inclusion and exclusion criteria, which ensured a well-characterized and homogenous patient cohort. Additionally, the detailed analysis of treatment outcomes and the integration of family dynamics into the evaluation process provide valuable insights into the psychosocial aspects of managing this condition. These factors enhance the generalizability and clinical relevance of our findings.

To summarize the clinical implications of our findings, we have created a table (Table 3) highlighting key aspects of early diagnosis and intervention strategies. This table consolidates the study’s contributions and provides actionable recommendations for clinical practice. Emphasis on family dynamics, the absence of generalized joint laxity, and the success of conservative management highlight the need for individualized, multidisciplinary approaches to managing this rare condition.

## 5. Conclusions

In conclusion, our findings suggest that integrating family therapy as a standard part of the treatment protocol may be highly relevant, particularly for patients from larger families. Addressing psychosocial factors through targeted family interventions could significantly enhance adherence to treatment and improve recovery outcomes.

This study represents Level III evidence, as it is a retrospective observational analysis. While the findings provide valuable insights, further prospective and multicenter studies are necessary to validate these results and refine treatment strategies. Future research should explore the use of dynamic imaging techniques, such as fluoroscopy or MRI during dislocation events, to better understand the biomechanics of voluntary habitual hip dislocations. These methods could provide valuable insights into the pathophysiology of the condition, potentially leading to more individualized treatment strategies.

## Figures and Tables

**Table 1:** Patient demographics, treatment methods, and follow-up outcomes.

Patients	Gender	Side	Age (Month)	Number of Children	Surgical Treatment	Orthosis	Recommendations and Follow-Up	Child Psychiatry	Child Psychiatry + Family Therapy	1 Month	6 Months	1 Year	2 Years
1. Patient	Male	Left	63	1	X		+			No dislocation	Dislocation	No dislocation	No dislocation
2. Patient	Male	Right	57	2	X		+			Dislocation	No dislocation	No dislocation	No dislocation
3. Patient	Male	Right	50	2	X			+		No dislocation	No dislocation	No dislocation	No dislocation
4. Patient	Female	Left	54	2	X		+			Dislocation	No dislocation	No dislocation	No dislocation
5. Patient	Female	Left	45	3	X			+		Dislocation	Dislocation	No dislocation	No dislocation
6. Patient	Female	Right	50	2	X			+		Dislocation	No dislocation	No dislocation	No dislocation
7. Patient	Female	Right	51	2	X			+		No dislocation	No dislocation	No dislocation	No dislocation
8. Patient	Female	Right	39	4	X	+	+			Dislocation	No dislocation	No dislocation	No dislocation
9. Patient	Female	Right	48	4	X			+		Dislocation	Dislocation	No dislocation	No dislocation
10. Patient	Female	Right	48	6	X			+	+	Dislocation	No dislocation	No dislocation	No dislocation
11. Patient	Female	Right	46	6	X			+	+	Dislocation	Dislocation	No dislocation	No dislocation
12. Patient	Female	Right	42	7	X	+		+	+	Dislocation	Dislocation	No dislocation	No dislocation
13. Patient	Female	Bilateral	41	8	X			+	+	Dislocation	Dislocation	No dislocation	No dislocation

**Table 2:** Treatment response rates by family group and follow-up periods.

Group	1st-Month Response Rate (%)	6th-Month Response Rate (%)	1-Year Response Rate (%)	2-Year Response Rate (%)	*p*-Value
Fewer than 3 children (Group 1)	90	95	100	100	0.033
3 or more children (Group 2)	60	70	100	100	0.048

**Table 3:** Implications for early diagnosis and intervention strategies.

Aspect	Key Findings/Implications	Recommendations
Clinical Presentation	Painless posterior dislocations without structural abnormalities.	Emphasize the role of detailed patient history and physical examination to identify habitual dislocations.
Role of Imaging	Standard imaging (X-ray, CT) reveals no structural abnormalities; dynamic imaging not feasible in this cohort.	Focus on clinical assessments for diagnosis; consider future use of advanced imaging techniques for research.
Generalized Joint Laxity	Absence of joint laxity (negative Beighton scores) highlights the behavioral nature of the condition.	Exclude systemic connective tissue disorders when joint laxity is absent.
Family Dynamics	Larger families associated with slower recovery due to reduced attention and psychological factors.	Integrate family therapy into treatment plans, particularly for larger families.
Conservative Management	High success rates (85% at 1 year, 100% at 2 years) with orthosis and family-focused interventions.	Prioritize conservative approaches as first-line treatment before considering surgical options.
Future Research Needs	Lack of data on surgical comparisons and long-term biomechanical insights.	Conduct prospective studies incorporating surgical comparisons and dynamic imaging for better understanding.

## Data Availability

The original contributions presented in the study are included in the article, and further inquiries can be directed to the corresponding author.
